# Application of the Sponge Model Implants in the Study of Vaccine Memory in Mice Previously Immunized with LBSap

**DOI:** 10.3390/vaccines12121322

**Published:** 2024-11-26

**Authors:** Mariana Ferreira Lanna, Lucilene Aparecida Resende, Paula Mello De Luca, Wanessa Moreira Goes, Maykelin Fuentes Zaldívar, André Tetzl Costa, Walderez Ornelas Dutra, Alexandre Barbosa Reis, Olindo Assis Martins-Filho, Kenneth Jhon Gollob, Sandra Aparecida Lima de Moura, Edelberto Santos Dias, Érika Michalsky Monteiro, Denise Silveira-Lemos, Rodolfo Cordeiro Giunchetti

**Affiliations:** 1 Laboratory of Biology of Cellular Interactions, Department of Morphology, Federal University of Minas Gerais, Belo Horizonte 31270-901, MG, Brazillucilenearesende@yahoo.com.br (L.A.R.);; 2 Immunopathology Laboratory, Núcleo de Pesquisas em Ciências Biológicas (NUPEB), Institute of Exact and Biological Sciences, Federal University of Ouro Preto, Ouro Preto 35400-000, MG, Brazil; 3 Instituto Oswaldo Cruz (IOC), FIOCRUZ Av. Brasil, Rio de Janeiro 21040-900, RJ, Brazil; 4 Integrated Biomarker Research Group, René Rachou Research Institute, Oswaldo Cruz Foundation, Belo Horizonte 30190-002, MG, Brazil; 5 Albert Einstein Israeli Institute of Education and Research, Albert Einstein Hospital, São Paulo 05652-900, SP, Brazil; 6 Biomaterials and Experimental Pathology Laboratory, Institute of Exact and Biological Sciences, Federal University of Ouro Preto, Ouro Preto 35400-000, MG, Brazil; 7 Taxonomy of Phlebotomines/Epidemiology, Diagnosis and Control of Leishmaniasis Group, René Rachou Research Institute, Oswaldo Cruz Foundation, Belo Horizonte 30190-002, MG, Brazil; edelberto.dias@fiocruz.br (E.S.D.); erika.monteiro@fiocruz.br (É.M.M.); 8 Department of Medicine, José Rosário Vellano University, Belo Horizonte Campus, Belo Horizonte 31270-020, MG, Brazil

**Keywords:** sponge implant model, vaccine, immune response, mice

## Abstract

Background/Objectives: Considering the large number of candidates in vaccine-testing studies against different pathogens and the amount of time spent in the preclinical and clinical trials, there is a pressing need to develop an improved in vivo system to quickly screen vaccine candidates. The model of a polyester–polyurethane sponge implant provides a rapid analysis of the specific stimulus–response, allowing the study of a compartmentalized microenvironment. The sponge implant’s defined measurements were standardized as a compartment to assess the immune response triggered by the vaccinal antigen. The LBSap vaccine (composed of *Leishmania braziliensis* antigens associated with saponin adjuvant) was used in the sponge model to assess the antigen-specific immunological biomarker, including memory generation after initial contact with the antigen. Methods: Mice strains (Swiss, BALB/c, and C57BL/6) were previously immunized using LBSap vaccine, followed by an antigenic booster performed inside the sponge implant. The sponge implants were assessed after 72 h, and the immune response pattern was analyzed according to leukocyte immunophenotyping and cytokine production. Results: After LBSap vaccination, the innate immune response of the antigenic booster in the sponge implants demonstrated higher levels in the Ly+ neutrophils and CD11c+ dendritic cells with reduced numbers of F4/80+ macrophages. Moreover, the adaptive immune response in Swiss mice demonstrated a high CD3+CD4+ T-cell frequency, consisting of an effector memory component, in addition to a cytoxicity response (CD3+CD8+ T cells), displaying the central memory biomarker. The major cell surface biomarker in the BALB/c mice strain was related to CD3+CD4+ effector memory, while the increased CD3+CD8+ effector memory was highlighted in C57/BL6. The cytokine profile was more inflammatory in Swiss mice, with the highest levels of IL-6, TNF, IFN-g, and IL-17, while the same cytokine was observed in in C57BL/6 yet modulated by enhanced IL-10 levels. Similar to Swiss mice, BALB/c mice triggered an inflammatory environment after the antigenic booster in the sponge implant with the increased levels in the ILL-6, TNF, and IFN-g. Conclusions: The findings emphasized the impact of genetic background on the populations engaged in immune responses, suggesting that this model can be utilized to enhance and track both innate and adaptive immune responses in vaccine candidates. Consequently, these results may inform the selection of the most suitable experimental model for biomolecule testing, taking into account how the unique characteristics of each mouse strain affect the immune response dynamics.

## 1. Introduction

The subcutaneous sponge implant model was originally described by Grindlay and Waugh (1951) [1] in dogs and by Edwards et al. (1960) [2] in rabbits as a framework for the growth of vascularized connective tissue, yielding valuable qualitative insights into neovascularization and wound healing. This model has since been utilized to quantify various biochemical parameters and to study cell proliferation kinetics in rats [3,4]. In 1987, Andrade and colleagues further modified the model for the quantitative study of in vivo angiogenesis by measuring blood flow in the implants as they vascularized. This methodology was successfully adapted in mice, allowing for the assessment of vascular development, biochemical variables, and the pharmacological reactivity of newly formed vessels [5].

Following this, the sponge implant has been applied as an important in vivo model for the study of angiogenesis and inflammatory processes [6,7,8,9]. After implantation in the subcutaneous compartment, the acellular and avascular sponge matrix induces the migration, proliferation, and accumulation of inflammatory cells, angiogenesis, and extracellular matrix deposition in its trabeculae [6,10]. The sponge implant model allows for the sequential study of the inflammatory infiltrate through histomorphometric and biochemical analyses, using the activity of the enzymes and myeloperoxidase (MPO) and N-acetyl-glucosaminidase (NAG) to indirectly determine the activation of neutrophils and macrophages [9,11,12,13].

Many studies investigate the compartmentalized microenvironment in response to implant-associated anti-inflammatory or anti-angiogenic molecules [7,10,14,15,16,17,18,19,20,21,22,23,24,25,26]. In the case of a vaccine capable of protecting against leishmaniasis, it is desirable that it induces a Th1-type immune response profile, where the pro-inflammatory cytokine IFN-γ/TNF must overcome the effects of the regulatory cytokine IL-10, while susceptibility is related to a deficient Th1 response [27]. These studies related to the development of vaccines also consider the use of animal models for experimental validation, as regulatory authorities require preclinical evaluations in these models before advancing to clinical trials [28].

Vaccine tests can utilize isogenic or heterogeneous animals. Heterogeneous models may better simulate diverse human populations [29], while isogenic strains ensure consistency in immunogenicity assessments, as shown by differences in immune responses among mouse lineages [30,31]. The BALB/c model is prevalent for testing against various microorganisms [30,32], but it may not accurately reflect the clinical course of certain diseases, as observed with dengue virus infections [33].

For anti-Leishmania vaccines, BALB/c mice are commonly used due to their susceptibility, unlike C57BL/6 mice, which produce a type I cytokine response. BALB/c mice infected with *L. infantum* show significant parasitic loads and hepatosplenomegaly, facilitating preclinical vaccine studies [34,35,36]. The characterization of the immune events, including leukocyte immunophenotyping, cell activation, and the cytokine profile in sponge implanted in mice (Swiss, BALB/c and C57BL/6), demonstrated the influence of the genetic background with the inflammatory kinetics [37]. The appropriate time points after the implant sponge as the platform of biomolecules screening was described according to the mice background: Day 5 post-implant for Swiss mice, Day 7 for BALB/c mice, and Day 6 for C57BL/6 mice. Those time points disclosed minor inflammation triggered by the sponge that induced an expected foreign body reaction post-implant [38]. Among the possible applications of studies using the sponge implant as a model, we highlight the possibility of trialing antigen candidates to compose protective vaccine formulations.

In this context, our group has described the ability of LBSap vaccine (composed by *Leishmania braziliensis* antigens associated with saponin adjuvant) to trigger a protective immune response in mice, hamsters, and dogs [39,40,41,42]. These studies supported the LBSap vaccine as a formulation presenting protective antigens useful for validating the sponge implant as model for memory immune response analyses. This new in vivo compartmentalized platform could be employed to optimize the screening of potential antigens required in protective vaccines. Thus, this study aimed to evaluate the sponge implant model in a vaccine memory test using the LBSap vaccine as a reference to improve the understanding of vaccine immunogenicity to establish a new model for in vivo tests. For this purpose, the immune response was further characterized using a prime (vaccine) and boost (antigen inoculation at sponge implant) protocol.

## 2. Materials and Methods

### 2.1. Animals

Male Swiss, BALB/c, and C57BL/6 mice (8–10 weeks old; *n* = 60 total, with 20 mice per strain) were obtained from the Centro de Ciências Animal (CCA) at the Federal University of Ouro Preto (UFOP), Brazil. The animals were housed in ventilated cages with ad libitum access to food and water throughout the study, under a 12-hour light/dark cycle. The experimental protocol was approved by the Animal Ethics Committee (CEUA/UFOP, #014/2011). Each strain was equally divided into four experimental groups, consisting of 5 mice per group.

### 2.2. Sponge Implants

All the procedures were performed as described by [38]. Briefly, disk-shaped (5 mm × 8 mm) polyether–polyurethane sponges were immersed overnight in 70% *v*/*v* ethanol and then boiled in distilled water for 15 min prior to implantation. Mice were anesthetized with an intraperitoneal injection of ketamine (150 mg/kg) and xylazine (10 mg/kg). The fur on the dorsal area was shaved, and the skin was disinfected with 70% *v*/*v* ethanol. Sponge discs were then subcutaneously implanted through a 1 cm dorsal midline incision. The animals were monitored daily for signs of discomfort, distress, or potential opportunistic infections.

### 2.3. Experimental Design

**Figure 1:**
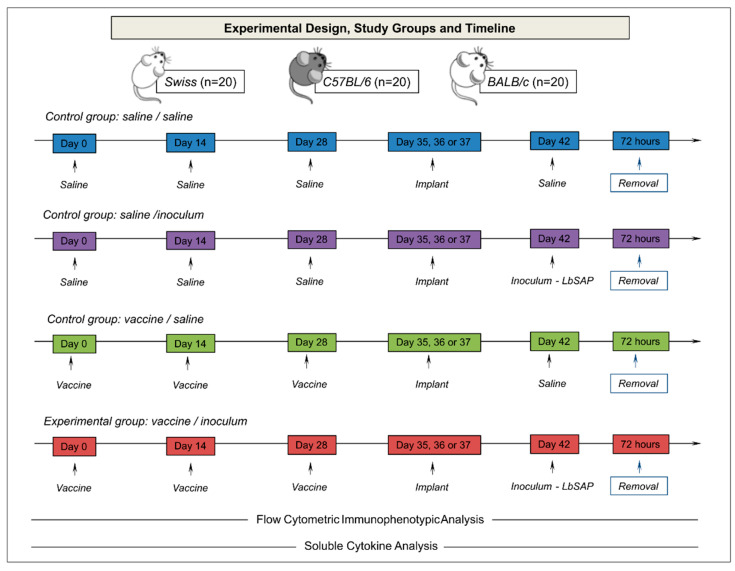
Experimental design and study groups. The animals were divided into three control groups and one experimental group, with a total of 5 mice per group. Statistical analyses were performed using ANOVA, followed by Tukey’s test. The implanted sponge discs were removed and sent for immunophenotyping analysis by flow cytometry and for investigation of soluble cytokines.

Previous research has indicated that the dynamics of phenotypic and functional alterations in the sponge microenvironment are essential for determining the optimal post-implantation timing, as this information is vital for conducting biomolecule screening assays [38]. Therefore, Day 5/Swiss, Day 7/BALB/c, and Day 6/C57BL/6 were chosen for an immunological memory study [38]. The sponge model was utilized to examine vaccine memory in animals that had been previously immunized. Details regarding the experimental setup, study groups, and timeline can be found in Figure 1.

The animals were divided into three control groups (Figure 1): saline/saline (S/S), which consisted of three saline inoculums, subcutaneously, with a 14-day interval and a new saline inoculum performed inside the sponge implant 14 days after the last inoculum; saline/inoculum (S/I), which consisted of three saline inoculums, subcutaneously, at 14-day intervals, and a new inoculum performed 14 days after the last inoculum in the sponge implant with 60 μg of *Leishmania braziliensis* antigen (produced as described by Giunchetti et al. [43]); vaccine/saline (V/S), which consisted of three inoculums of LBSap vaccine (60 μg of *Leishmania braziliensis* antigen and 50 μg saponin per dose—produced as described by Giunchetti et al. [43]), subcutaneously, with a 14-day interval; a new inoculum was performed 14 days after the last inoculum in the sponge implant with saline; and an experimental group vaccine/inoculum (V/I), which consisted of three inoculums of the LBSap vaccine (60 μg of *Leishmania braziliensis* antigen and 50 μg saponin per dose), subcutaneously, at 14-day interval and a new inoculum performed 14 days after the last inoculum on the sponge implant with 60 μg of *Leishmania braziliensis* antigen. After 72 h of the different inoculum in the sponge implants, the animals were euthanized, and the sponges were removed for immunophenotypic analysis and soluble cytokine measurements.

### 2.4. Flow Cytometry Immunophenotyping

The leukocyte subsets were harvested from sponge implants and analyzed by flow cytometry, as previously described Lanna et al. [38]. A panel of fluorescent monoclonal antibodies was used, including anti-CD45 (APC clone 30-F11/E071491630, FITC clone Sa230-F11/E003051630), anti-CD3 (Pe-Cy5 clone 145-2C11/E060661630), anti-CD8 (APC clone 53.6-7/E070561330), anti-CD11c (Pe-Cy5 clone N418/E006121631), F4/80 (FITC clone BM8/E006121631), LY6G (APC clone RB6-8C5/E001610630), anti-CD197 (PerCPCy5.5 clone 4B12/E0800369258) and anti-CD62L (Pe clone MEL-14/E009159357) from e-Bioscience (San Diego, CA, USA), and anti-CD4 (FITC clone RM4-5/714474A) from Invitrogen (Carlsbad, CA, USA).

Immunophenotypic analysis was performed to assess leukocyte types and lymphocyte subtypes. Initially, a gate encompassing all CD45+ cells was selected and displayed on a graph for the analysis of leukocyte types (Ly+ neutrophils, F4/80+ macrophages, CD11c+ dendritic cells, and CD3+ lymphocytes; all of which are CD45+). Among the acquired immune cells, a second gate was applied to study the lymphocyte subpopulations (CD3+CD4+ and CD3+CD8+). Specifically, among the CD45+CD3+ cells, the CD3+CD4+ and CD3+CD8+ subpopulations were separated. Subsequently, each of these subpopulations was further analyzed to generate a new gate for exploring memory cells (central memory (CM) CD62L+CCR7+ and effector memory (EM) CD62L-CCR7-) within both the CD3+CD4+ and CD3+CD8+ subpopulations.

The sponges were extracted from the implantation site and incubated for one hour in a trypsin solution. Afterward, the sponge implants were gently compressed at room temperature in 2 mL of RPMI, and the sponge debris was removed by differential centrifugation at 200× *g* for 5 min at 4 °C. The supernatant was then centrifuged at 400× *g* for 8 min at 4 °C to collect the cell pellet. Following resuspension of the cell pellet, erythrocytes were lysed using 10 mL of ammonium chloride buffer. The leukocytes were washed once with 10 mL of RPMI at 400× *g* for 8 min at 4 °C. Cell counts were determined using a Neubauer chamber, and the final cell suspension was adjusted to 1 × 10^5^ cells/mL. Aliquots of 100 μL of the cell suspension (containing approximately 1 × 10^4^ cells per tube) were incubated in polypropylene tubes with 20 μL of monoclonal antibody combinations. After incubation, the stained cells were washed once with phosphate-buffered saline (PBS) and resuspended in 250 μL of PBS. Nonspecific binding was assessed using fluorochrome-labeled, isotype-matched reagents to provide appropriate negative controls. Autofluorescence was evaluated using a negative control, in which the cell suspension was incubated without fluorochrome-labeled monoclonal antibodies but with dilution and wash buffers. Flow cytometric analysis was performed on a FACSCanto II^®^ instrument (Becton Dickinson, Mountain View, CA, USA), with 20,000 events acquired per sample. Data acquisition and storage were carried out using CELLQuestPro software (Franklin Lakes, NJ, USA), and FlowJo Software (Version 10.1, Tree Star, Inc., Ashland, OR, USA) was used for data analysis.

### 2.5. Assessment of Soluble Cytokine Levels

Sponge implants were homogenized in 1 mL of RPMI, using a tissue homogenizer (Homo mix). The homogenates were then centrifuged at 3000× *g* for 10 min at 4 °C, and the supernatants were stored at −80 °C until further processing. The levels of soluble cytokines were assessed using a Cytometric Bead Array (BD Biosciences, San Jose, CA, USA), following the manufacturer’s instructions. The mouse inflammation kit was employed to quantify soluble IL-6, IL-10, IL-17, IFN-γ, and TNF levels. Data analysis was conducted using FCAP software v.1.0.2 (BD Biosciences), and results were reported as median fluorescent intensity (MFI) values.

### 2.6. Statistical Methods

Intragroup and intergroup comparative analysis were performed by one-way ANOVA, followed by Tukey’s multiple comparison test, to compare all pairs of data. The normality of the data in all groups was assessed using the Shapiro–Wilk test, which confirmed that the majority of datasets followed a normal distribution, justifying the use of parametric tests. In all cases, significance was considered at *p* < 0.05. GraphPad Prism (version 5.03, San Diego, CA, USA) was used for statistical analysis and graphic arts.

### 2.7. Evaluation of Biomarker Signatures

The analysis of biomarker signatures was conducted following the methodology described by Luiza-Silva et al. that emphasizes cytokine profiles in both innate and adaptive immunity following yellow fever vaccination [44]. This approach enables the detection of subtle variations that are often missed by traditional statistical methods yet are crucial for comprehending the intricate immunological microenvironments that encompass multiple events.

Biomarker signatures were established by evaluating the frequency of implants with biomarker levels surpassing the global median cutoff defined for each biomarker. To determine this global median cutoff, the dataset was sorted in ascending order, and the median was computed for each subset of cells (CD45+, Ly+, F4/80+, CD11c+, CD3+CD4+, CD3+CD8+, CD62L+CCR7+, and CD62L-CCR7-), as well as for each soluble cytokine (IL-6, TNF, IFN-γ, IL-10, and IL-17), using Microsoft Excel software. This analysis incorporated data from all indicated biomarkers, including information from all experimental groups (S/S, S/I, V/S, and V/I) and all three mouse strains (Swiss, BALB/c, and C57BL/6).

The results were classified based on the global median cutoff, with values categorized as “low” (below the global median) or “high” (above the global median). These categorical data were then utilized to calculate the proportion (percentage) of implants exhibiting biomarker levels above the global median. A curve representing the ascending frequencies of the biomarkers was generated for each experimental group. Biomarkers with frequencies exceeding 75% were deemed significant and highlighted in bold/underlined format. Microsoft Excel software was employed to create radar charts and finalize the graphical representations.

## 3. Results

**Figure 2:**
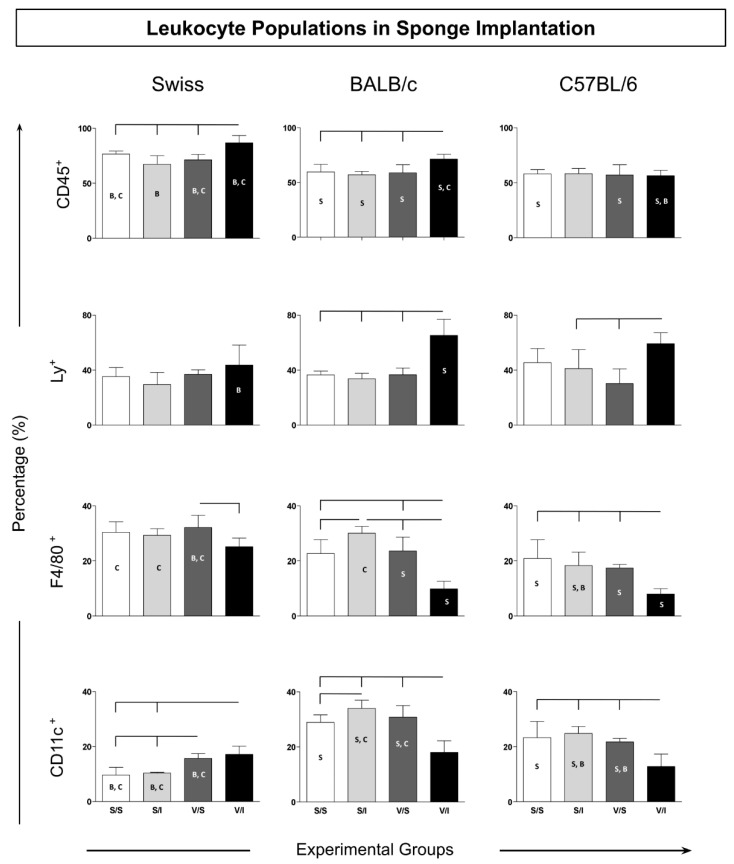
Immunophenotypic profile of leukocytes in sponge implants. The cellular infiltrate removed from the implants was labeled with a monoclonal antibody for quantification by flow cytometry of leukocyte subpopulations (CD45+) of innate immunity cells: neutrophils (LY+), macrophages (F4/80+), and dendritic cells (CD11c+). Data are reported as mean percentage ± standard deviation (*n* = 5 mice per group). Intragroup and intergroup comparisons were evaluated using ANOVA, followed by Tukey’s test, and significant differences (*p* < 0.05) are highlighted by connecting lines within the lineage and by the letters “S”, “B”, and “C” for comparisons between Swiss, BALB/c, and C57BL/6 mice, respectively.

The data presented in Figure 2 illustrate the innate immune response characterized by the populations of total CD45+ leukocytes and specific subpopulations, including dendritic cells (CD11c+), macrophages (F4/80+), and neutrophils (Ly+).

In Swiss mice, a significant increase in the total CD45+ leukocyte population was observed in the experimental group (V/I) compared to the control groups (S/S, S/I, and V/S), as well as in relation to BALB/c mice under the same conditions (S/S and S/I). This indicates a robust immune activation in response to the experimental treatment, highlighting the heightened capacity of Swiss mice to mobilize leukocytes upon antigen exposure.

Regarding neutrophils (Ly+), BALB/c mice showed a marked increase in the V/I group relative to other groups, suggesting a strong neutrophilic response to vaccination. Similarly, C57BL/6 mice exhibited higher percentages of Ly+ neutrophils in the V/I group compared to the S/I and V/S groups, while Swiss mice demonstrated a lower percentage of Ly+ neutrophils in the V/I group when compared to BALB/c mice, indicating divergence in neutrophil recruitment or activation pathways among strains. The population of F4/80+ macrophages exhibited a heterogeneous distribution, with lower percentages in the V/I group across all strains, suggesting potential modulation of macrophage activity in response to the vaccine.

Furthermore, an increase in CD11c+ dendritic cells was noted in Swiss mice in the V/S and V/I groups compared to the control groups (S/S and S/I), indicating enhanced dendritic cell activation in response to the vaccine. Conversely, BALB/c and C57BL/6 mice showed a decrease in CD11c+ dendritic cells in the V/I group, suggesting the possible suppression of dendritic cell function. Overall, these findings highlight distinct patterns of leukocyte responses across different mouse strains, reflecting inherent differences in immune functionalities and suggesting strain-specific adaptations to immunization strategies.

**Figure 3:**
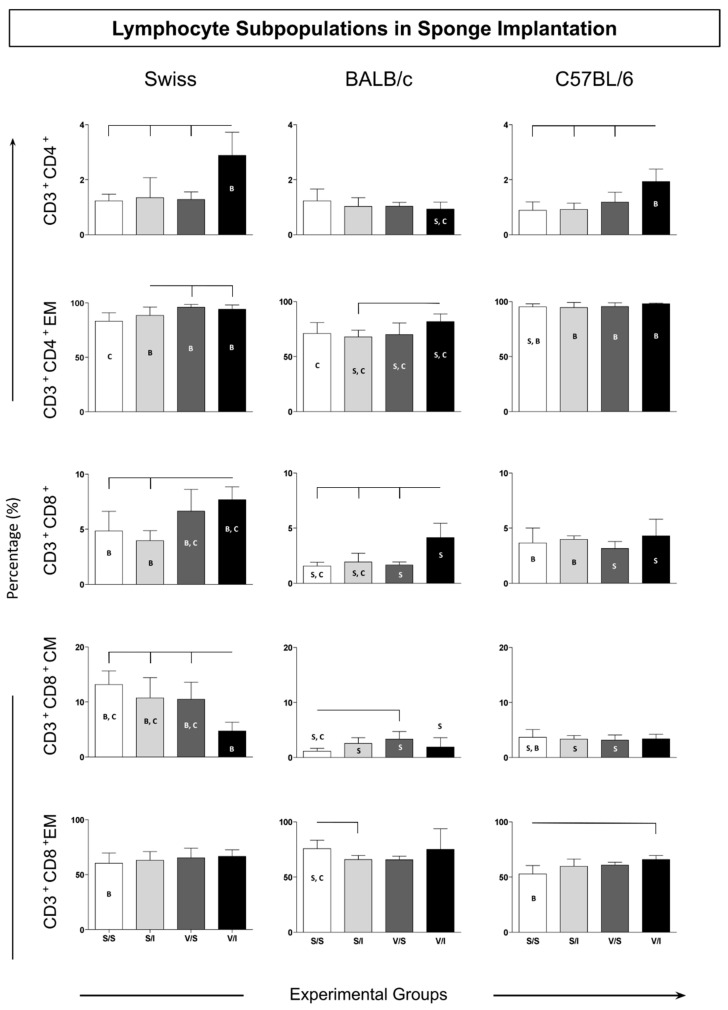
Immunophenotypic profile of lymphocytes in sponge implants. The cellular infiltrate was quantified by flow cytometry for the TCD3+ CD4+ and TCD3+ CD8+ lymphocyte subpopulations, in addition to the EM effector memory (CD62L+CCR7+) and CM central (CD62L+CCR7+) subpopulations. Data are reported as mean percentage ± standard deviation (*n* = 5 mice per group). Intragroup and intergroup comparisons were evaluated using ANOVA, followed by Tukey’s test, and significant differences (*p* < 0.05) are highlighted by connecting lines within the lineage and by the letters “S”, “B”, and “C” for comparisons between Swiss, BALB/c, and C57BL/6 mice, respectively.

The adaptive immune response cells were evaluated according to the total T cells (CD3+) and their T-cell subsets (CD3+CD4+ and CD3+CD8+). Also analyzed in those T-cell subsets were the immunological memory biomarkers: CD62L+CCR7+ (central memory) and CD62L-CCR7- (effector memory) (Figure 3). The data described a similar pattern of CD3+CD4+ T cells in Swiss and C57BL/6 mice with increased counts in the V/I group as compared to the other experimental groups (S/S, S/I, and V/S) (Figure 3—upper panel). In contrast, the BALB/c mice presented the lowest levels in the CD4+ T cells in the V/I group as compared to the other two mice strains (Swiss and C57/BL6) (Figure 3). Despite the effector memory (EM) CD4+ T cells, the Swiss mice presented enhanced levels in the V/S and V/I groups as compared to the S/S group (Figure 3—middle panel). Similar results were observed in the BALB/c mice, showing higher counts of EM CD4+ T cells in the V/I group as compared to the S/I group. However, in the C57BL/6, all analyzed groups displayed almost one hundred percent of the CD4+ T-cell EM.

The CD8+ T-cell frequency in Swiss mice showed increased levels in the V/I group as compared to the S/S and S/I groups (Figure 3—middle panel). The V/I group in BALB/c mice also exhibited an increase as compared to the other three groups (S/S, S/I, and V/S). Although no changes had been observed in the groups of the C57BL/6 mice, the levels of CD3+CD8+ T cells were the highest in the groups V/S and V/I of Swiss mice as compared to the same groups from BALB/c and C57/BL6. The central memory (CM) CD8+ T cells in Swiss mice showed lower levels in the V/I group as compared to the other experimental groups (Figure 3—middle panel). While there was no change in the CM CD8+ T cells between the groups of C57BL/6 mice, enhanced counts were observed in the V/S group of BALB/c mice as compared to the S/S group. The Swiss mice presented the highest levels of CM CD8+ T cells in relation to the other mice strains. Concerning effector memory (EM) CD8+ T cells, the BALB/C mice displayed a decrease in the S/I group as compared to the S/S group, whereas in C57BL/6 mice, the V/I group showed higher levels of this cell population as compared to the S/S group (Figure 3—bottom panel).

In summary, the observed increases in CD8+ T-cell populations, particularly in Swiss and BALB/c mice, may be closely related to the enhanced activation of dendritic cells (CD11c+) noted in Swiss mice. The increased CD11c+ dendritic cell population in the V/S and V/I groups indicates a potent antigen-presentation capability, which likely plays a critical role in priming CD8+ T cells. This is especially relevant given that dendritic cells are key facilitators of T-cell activation and differentiation, and their presence can significantly influence the quality of the adaptive immune response.

Furthermore, the differences in the central memory (CM) CD8+ T-cell levels between strains suggest that the immune system’s memory formation may vary based on the genetic background of the mice. For instance, Swiss mice demonstrated the highest levels of CM CD8+ T cells compared to BALB/c and C57BL/6 strains, indicating a potentially more robust memory response that could be beneficial for long-term immunity following vaccination.

Additionally, the relationship between the populations of F4/80+ macrophages and CD8+ T cells may also be worth exploring. While the percentages of macrophages were lower in the V/I group across all strains, understanding how these cells interact with T cells and dendritic cells could provide further insights into the mechanisms underlying the immune response in this model.

Overall, the integration of these findings underscores the complex interplay between different immune cell populations and highlights the importance of the sponge implant model for studying vaccine responses. Future investigations should focus on quantifying the immune response more rigorously and exploring the interactions between dendritic cells, T cells, and macrophages to elucidate the mechanisms driving immune activation in various strains.

### 3.1. Cytokine Microenvironment in the Sponge Implants

**Figure 4:**
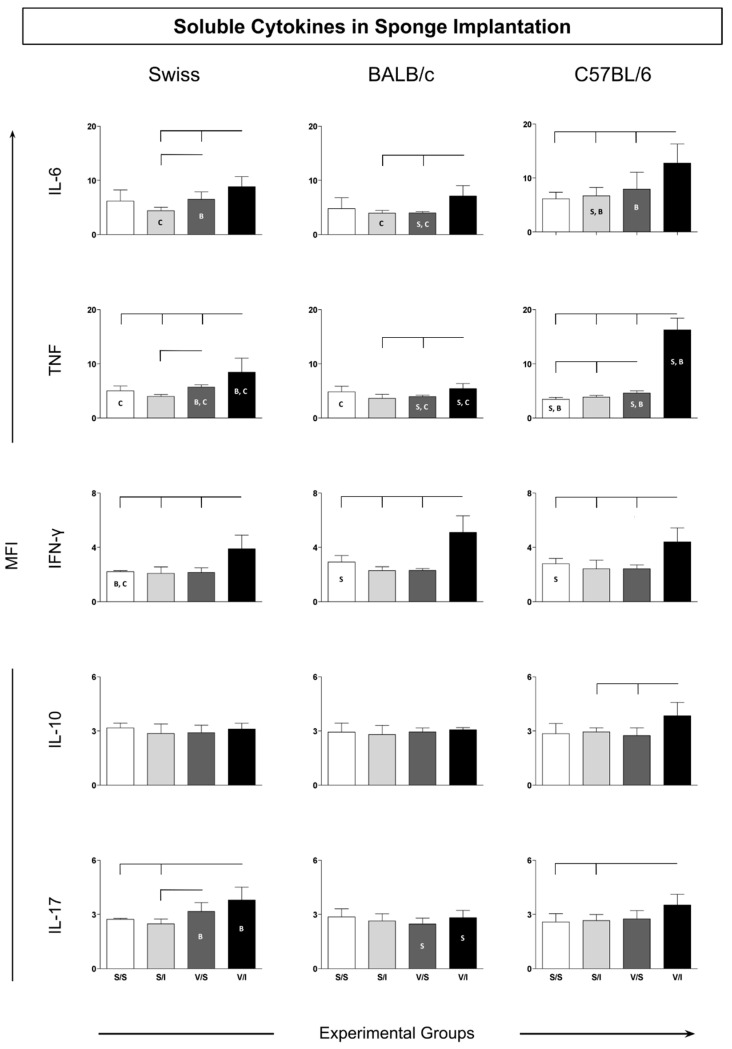
Soluble cytokines in the microenvironment of sponge implants. Data are reported as mean fluorescence intensity (MFI) ± standard deviation for each cytokine (IL-6, TNF, IFN-γ, IL-10, and IL-17) (*n* = 5 mice per group). Significant differences (*p* < 0.05) were evaluated using ANOVA, followed by Tukey’s test, and are represented by connecting lines above the graphs (differences considering the same lineage but different groups) and by the letters “S”, “B”, and “C” for comparisons among Swiss, BALB/c, and C57BL/6 mice, respectively.

The cytokine microenvironment in the sponge implants was analyzed to determine the amounts of soluble cytokines (IL-6, TNF, IFN-γ, IL-10, and IL-17). This panel of cytokines was chosen to evaluate the induction of a microenvironment related to an inflammatory or immunomodulatory profile in the microenvironment of the sponge implant. The soluble cytokines in the sponge implant for Swiss, BALB/c, and C57/BL6 mice strains showed higher IL-6 levels in the V/I group as compared to the S/I and V/S groups (Figure 4—upper panel). There was also an increase in TNF levels in the V/I groups when considering all mice strains when compared to the other experimental groups.

Notably, the V/I group in C57BL/6 mice displayed the highest IL-6 levels as compared to Swiss and BALB/c mice strains. Similarly, an increase in IFN-γ levels in all the mice strains was observed for V/I group as compared to the other three experimental groups (S/S, S/I, and V/S; Figure 4—middle panel). The IL-10 cytokine analysis revealed increased levels in C57BL/6 mice when considering the V/I group as compared to the S/I group (Figure 4—middle panel). Similar to other inflammatory cytokines, the IL-17 profile demonstrated higher levels in both Swiss and C57BL/6 mice in the V/I group as compared to the S/S and S/I groups without changes in the groups of BALB/c mice (Figure 4—bottom panel).

### 3.2. Ascendant Biomarker Signature Implants

**Figure 5:**
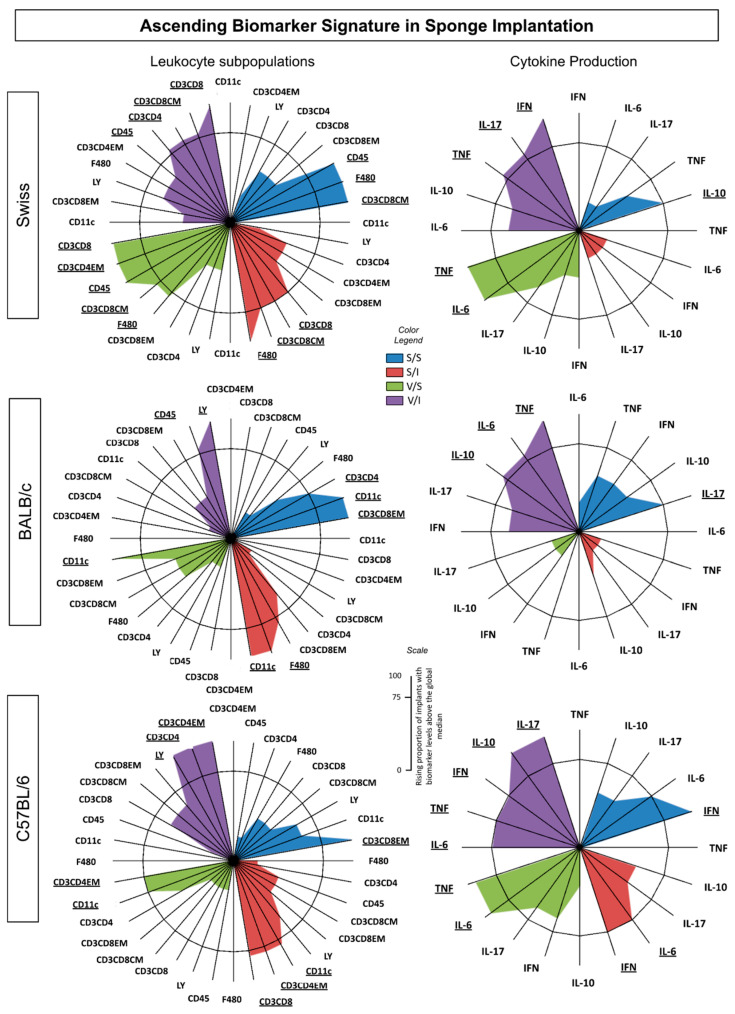
Ascending biomarker signature in sponge implants. This figure illustrates the frequency of biomarker levels above the global median cutoff for each cell subset (CD45+, Ly+, F4/80+, CD11c+, CD3+CD4+, CD3+CD4+ EM, CD3+ CD8+, CD3+CD8+ CM, and CD3+CD8+ EM) and soluble cytokines (IL-6, TNF, IFN-γ, IL-10, and IL-17) across different experimental groups: S/S (blue), S/I (red), V/S (green), and V/I (purple) for Swiss, BALB/c, and C57BL/6 mice (*n* = 5 mice per group). Significant differences (*p* < 0.05) were evaluated using ANOVA, followed by Tukey’s test. Biomarkers with frequencies equal to or above 75% were considered relevant and are highlighted in underlined bold format for emphasis.

To delineate the immunophenotypic and functional profiles of the immune response in the sponge across various experimental groups, the ascending biomarker signatures (arranged from lowest to highest frequency) were compiled, as illustrated in Figure 5. The data analysis validated the majority of the results identified through conventional statistical methods. The ascending biomarker signature within sponge implants consists of two categories of data: leukocyte (LEU) subsets and cytokine production.

Swiss mice exhibited the most prominent immune response to the sponge implant, vaccine, and inoculum (Figure 5). This strain displayed higher levels of cell surface biomarkers across all four groups, indicating robust recruitment of cytotoxic and central-memory immune responses (Figure 5—upper panel). Notably, Swiss mice demonstrated an intense inflammatory profile characterized by the highest frequencies of TNF, IL-17, and IFN-γ, with little modulation by IL-10, suggesting a strong inflammatory response driven by the vaccine and the sponge implant itself. In contrast, BALB/c mice showed a lower number of cell surface biomarkers, along with a cytokine environment primarily composed of inflammatory cytokines (TNF and IL-6) that were modulated by IL-10 (Figure 5—middle panel). This indicates a more regulated immune response compared to Swiss mice, reflecting the strain’s inherent characteristics in handling inflammation.

Furthermore, C57BL/6 mice exhibited a pronounced T helper-cell surface biomarker with an effector memory component, showcasing a significant inflammatory environment with the highest frequencies of IL-6, TNF, and IFN-γ, again modulated by IL-10 (Figure 5—bottom panel). Importantly, the results also indicate that the vehicle used in the S/I group may have had immunological effects, as evidenced by the differences observed between the S/I group and the other experimental groups. Overall, these findings underscore the distinct immune profiles of different mouse strains and the varying effects of the sponge implant and vaccine, emphasizing the complexity of the immune response and suggesting that strain-specific adaptations play a significant role in the effectiveness of vaccination strategies.

## 4. Discussion

The sponge model has been widely studied over the past three decades [7,10,13,14,16,19,20,45,46,47,48,49,50,51,52,53], including the phenotypic and functional changes that are triggered in distinct mouse lineages [38]. However, the use of this model for trialing antigen candidates for vaccine formulation has not yet been explored as a tool for determining the immunogenicity profile. The present study aimed to evaluate the sponge implant model for improving the understanding of immunogenicity to establish a new model of antigen in vivo testing. The time points for the immune response analysis in the sponge implants were chosen based on the data previously presented for each mouse strain [38].

This study described the changes that vaccination and/or antigen inoculation inside the sponge induced in the total leucocytes (CD45+) and in the distinct leukocyte subsets harvested from the sponge, in addition to the cytokine environment. It is important to emphasize that an immune response to an antigen can be determined by the environment in which it is found, being modulated by molecules such as cytokines [54,55]. That is, the formation of a microenvironment with different cytokines can effectively direct an immune response [56]. For example, the production of IFN-γ plays an essential role in the induction of antiparasitic responses in macrophages, notably by inducing the production of reactive oxygen species (ROS) and nitric oxide synthase (iNOS), necessary for the intracellular killing of Leishmania [57]. In the case of a vaccine capable of protecting against leishmaniasis, it is desirable that it induces a Th1-type immune response profile, where the pro-inflammatory cytokines IFN-γ/TNF must overcome the effects of the regulatory cytokine IL-10, while susceptibility is related to a deficient Th1 response [27]. The findings showed an increase in the levels of CD45+ leukocytes in the prime–boost protocol (related to group V/I) in two (Swiss and BALB/c) of the mice strains. In Swiss mice, the higher CD45+ leukocyte counts could be explained by the increase in CD11c+ dendritic cells and in T-cell subsets (CD3+CD4+ and CD3+CD8+). Surprisingly, the vaccination in BALB/c and C57BL/6 mice, followed by an antigenic boost in the sponge implant (group V/I), showed an increase in the Ly+ neutrophils, while exhibiting a reduction in the levels of CD11+ dendritic cells and F4/80+ macrophages. Furthermore, the increase in Ly+ neutrophils could partially explain the higher percentage of leukocytes in BALB/c. Interestingly, Mendonça et al. observed a reduction in the frequency of neutrophils when analyzing peripheral blood in BALB/c mice 15 days after LBSap vaccination [39]. These results could indicate the migration of neutrophils to the inflammation (vaccine) site triggered in BALB/c mice. Those results highlighted how the genetic background could influence the immune response triggered by the prime (vaccine) and boost (antigen inoculation at sponge implant) protocol. The analysis of F4/80+ macrophages demonstrated a similar pattern of cell infiltration, since the percentage of these cells in all mice strains was lower as a result of the prime–boost protocol (group V/I). In addition, in BALB/c and C57BL/6 mice, the decreased levels of CD11+ dendritic cells after the prime–boost protocol (group V/I) could be explained by the migration to lymphoid organs, such as lymph nodes, for antigen presentation [58]. It is important to emphasize that, in BALB/c mice, increases in both F4/80+ macrophages and CD11+ dendritic cells were observed in the S/I group. It is possible to hypothesize that the type of inoculum (presence of the antigen), without prior vaccination, could temporally trigger this stimulus since both cell types are specialized in antigen presentation.

An investigation of the adaptive immune response showed that the prime–boost protocol was enough to stimulate the selective migration of CD3+CD4+ T cells in Swiss and C57BL/6, but not in BALB/c mice. It is important to emphasize that the induction of an increase in the amount of CD3+CD4+ T cells by anti-visceral leishmaniasis vaccines has been associated with high levels of protection due to the ability of these lymphocytes to produce IFN-γ and promote the activation of macrophages favoring leishmanicidal activity [59]. In contrast, a higher count of CD3+CD8+ T cells was elicited in the BALB/c mice but at a lower level as compared to Swiss mice (V/I group). The previous vaccination of Swiss mice was effective in increasing the levels of CD3+CD8+ T cells, while the association with the boost (antigen inoculum in the sponge) was required in BALB/c for the expansion of this T-cell subset. Additionally, it has been reported that the LBSap immunization is able to induce enhanced frequency in the circulating T cells, including their subsets (CD4+ and CD8+) [43]. Notably, the levels of CD4+ and CD8+ T cells remained high after the *L. infantum* experimental challenge, and it was associated with the protection profile [60].

The innate and adaptive immune responses may be related to activation events and the maintenance of a protective response profile after vaccination. However, the adaptive response allows the host to trigger an immune memory response, which is of paramount importance in an effective response against pathogens [56,61]. The expansion phase and effector phase of the immune response are followed by an apoptosis phase, during which most antigen-specific T cells perish. The final stage, known as the memory phase, is marked by the persistence of long-lived memory T cells that retain their specificity for a particular antigen and can be sustained throughout the host’s lifetime [62,63]. A key distinguishing feature of a memory immune response, compared to a primary response, is its ability to swiftly produce larger quantities of antigen-specific helper T cells and cytotoxic agents. While memory T cells do not prevent reinfection or disease recurrence, they mount a rapid and efficient response in these situations, which is a fundamental mechanism behind the effectiveness of many vaccines [56,58]. Memory T cells can be classified based on their migratory capabilities, proliferative potential, and ability to produce cytokines [64,65]. Briefly, effector memory (EM) T cells (CD62L-CCR7-) are present in non-lymphoid peripheral tissues and also circulate in the blood. They can migrate through tissues in search of a particular antigen, where they quickly exert effector functions, such as IFN production and cytotoxic activity [63,64]. However, despite existing in large numbers, effector memory T cells do not have proliferative potential. Thus, a second line of defense is constituted by central memory (CM) cells (CD62L+CCR7+), which reside and circulate among the secondary lymphoid organs. These cells are type 2 interleukin producers and are very sensitive to antigenic stimulation, capable of rapid proliferation [58].

In summary, EM T cells provide protection against reinfection or disease reactivation at infection sites, whereas CM T cells primarily reside in the lymphoid tissue, where they rapidly expand and differentiate to replenish the population of effector T cells. The results of this study demonstrated that Swiss mice are able to trigger an immune response after prime–boost (V/I group) using *Leishmania* antigens marked by (i) increased CD3+CD4+ T cells and (ii) CD3+CD8+ T cells with (iii) effector memory based on CD3+CD4+. In BALB/c mice, this prime–boost antigenic response was based on (i) sustained levels of CD3+CD4+ with (ii) induction in effector memory CD3+CD4+ T cells and (iii) a higher frequency of CD3+CD8+ T cells. This immune response in C57BL/6 was marked by (i) increased levels in the CD3+CD4+ T cells, (ii) sustained levels of effector memory CD3+CD4+ T cells, and (iii) enhanced frequency of effector memory CD3+CD8+ T cells. In fact, previous data have reported that the LBSap vaccine presented a higher immunogenicity pattern displaying higher levels in the T-cell subsets (CD4+ and CD8+) [43], resulting in improved protection against *L. infantum* infection [39,60,66,67,68]. Additionally, the sponge implant model was able to further characterize the immune response after the prime–boost protocol, providing different mechanisms associated with protection described in the LBSap vaccine in Swiss mice.

Our functional analysis of the sponge microenvironment revealed that C57BL/6 mice induced a more prominent response after the prime–boost protocol (group V/I) as compared to the other groups, presenting higher levels of cytokines with a mixed activation profile (IL- 6, TNF, IFN-γ, IL-10, and IL-17). In contrast, the cytokine analysis in Swiss mice revealed a prominent inflammatory response mediated mainly by IL-6, TNF, IFN-γ, and IL-17. Additionally, the BALB/c mice showed a similar inflammatory cytokine environment but confined to IL-6, TNF, and IFN-γ. It is important to emphasize that the immune response to an antigen can be determined by the environment in which it is found, being modulated by molecules such as cytokines [54,55]. In fact, the antigen in this microenvironment can trigger a different cytokine production that effectively drives the pattern of the immune response [56]. For example, the production of IFN-γ plays an essential role in inducing antiparasitic responses in macrophages, notably inducing the production of reactive oxygen species (ROS) and nitric oxide synthase (iNOS), essential for killing intracellular pathogens, such as *Leishmania* [57]. In addition, infection control in visceral leishmaniasis has been related to a Th1-type immune response, in which the pro-inflammatory cytokines IFN-γ/TNF must overcome the effects of the regulatory cytokine (IL-10), while susceptibility is related to a deficient pro-inflammatory response [27,66,68,69]. Some studies also report the importance of the cytokine IL-17 in controlling visceral leishmaniasis and developing vaccines against the disease [70]. According to Pitta et al. and Nascimento et al., this cytokine plays an important protective role in human visceral leishmaniasis, complementing the protection conferred by inflammatory cytokines in a non-dependent manner or in synergy with IFN-γ, enhancing its action [71,72]. Similar to the data on cellular immune response described in Swiss mice, this strain presented a predominantly pro-inflammatory cytokine profile, reinforcing the applicability of this mice strain for an immunogenicity analysis of vaccinal antigens. These data are supported by LBSap studies, demonstrating the presence of a pro-inflammatory cytokine profile (such as IL-6, TNF, IFN-γ; and an increased IFN-γ/IL-10 rate) associated with protection in visceral leishmaniasis [39,59,66,68].

## 5. Conclusions

The data from this study demonstrated that the implant using a polyester–polyurethane sponge in distinct mice strains provides a reliable in vivo model for immunogenicity studies applied to antigen testing for vaccine candidates. The data described the approach for immunogenicity testing using prime (vaccination) and boost (antigen inoculation at sponge implant), and this approach was able to trigger the innate and adaptive immune responses. The selective stimulation in Ly+ neutrophils and CD11c+ dendritic cells reinforced the ability to assess the innate immune response. Moreover, the adaptive immune response was also induced, as demonstrated by the induction in antigen-specific T-cell subsets, corroborating the application of this approach for immunogenicity testing of vaccine (antigens) candidates. In fact, the differential induction on T-cell subsets (CD4+ and CD8+) in the distinct mice strains validated the ability of this model to elicit antigen-specific central and effector memory immune responses. The Swiss mice presented the primary elements for immunogenicity analysis (induction of central memory based on T-cell cytoxicity and an intense inflammatory profile compose by IFN-γ, IL-17, and TNF). Although the BALB/c mice were not marked by a significant influence of adaptive immune response (T-cell memory), the microenvironment elicited in the sponge implant demonstrated a mixed pattern (with inflammatory (TNF, IL-6) and immunomodulatory (IL-10) cytokines). The C57/BL6 mice showed immune biomarkers related to both Swiss and BALB/c mice, with a prominent amount of CD4+ T cells (including effector memory CD4+ T cells) and an intense production of inflammatory cytokines (IL-17, IFN-γ, TNF, and IL-6) modulated by IL-10. The results highlighted how genetic background can influence the populations involved in the immune response and indicate that this model could be used to monitor the innate and adaptive immune responses of candidate vaccines. In this sense, these results could guide the choice of the most appropriate experimental model for testing biomolecules, given that the particularities of each mouse strain influenced the dynamics of the innate and adaptive immune responses.

## Data Availability

All datasets generated for this study are included in the manuscript.
